# Effect of nicotine on *Staphylococcus aureus* biofilm formation and virulence factors

**DOI:** 10.1038/s41598-019-56627-0

**Published:** 2019-12-27

**Authors:** Le Shi, Yang Wu, Chen Yang, Yue Ma, Qing-zhao Zhang, Wei Huang, Xiao-yi Zhu, Ying-jie Yan, Jia-xue Wang, Tao Zhu, Di Qu, Chun-quan Zheng, Ke-Qing Zhao

**Affiliations:** 1 Department of Otorhinolaryngology and Head and Neck Surgery, Eye & ENT Hospital, Shanghai Key Clinical Disciplines of Otorhinolaryngology, Shanghai Medical College, Fudan University, Shanghai, P.R. China; 2 Key Laboratory of Medical Molecular Virology (MOE/NHC/CAMS), Department of Medical Microbiology and Parasitology, School of Basic Medical Sciences, Shanghai Medical College, Fudan University, Shanghai, P.R. China; 3 Department of Otolaryngology & Head and Neck Surgery, Ruijin Hospital, Shanghai Jiao Tong University School of Medicine, Shanghai, P.R. China; 4 Medical Clinic, Hangzhou Haiqin Sanatorium, Hangzhou, Zhejiang P.R. China; 5 Department of Laboratory Medicine, Hangzhou Medical College, Hangzhou, Zhejiang P.R. China; 6 Department of Preclinical Medicine, Wannan Medical College, Wuhu, P.R. China

**Keywords:** Biofilms, Pathogenesis

## Abstract

*Staphylococcus aureus* is a common pathogen in chronic rhinosinusitis (CRS) patients, the pathogenesis of which involves the ability to form biofilms and produce various virulence factors. Tobacco smoke, another risk factor of CRS, facilitates *S. aureus* biofilm formation; however, the mechanisms involved are unclear. Here, we studied the effect of nicotine on *S. aureus* biofilm formation and the expression of virulence-related genes. *S. aureus* strains isolated from CRS patients and a USA300 strain were treated with nicotine or were untreated (control). Nicotine-treated *S. aureus* strains showed dose-dependent increases in biofilm formation, lower virulence, enhanced initial attachment, increased extracellular DNA release, and a higher autolysis rate, involving dysregulation of the accessory gene regulator (Agr) quorum-sensing system. Consequently, the expression of autolysis-related genes *lytN* and *atlA*, and the percentage of dead cells in biofilms was increased. However, the expression of virulence-related genes, including *hla*, *hlb*, *pvl*, *nuc*, *ssp*, *spa*, *sigB*, *coa*, and *crtN* was downregulated and there was reduced bacterial invasion of A549 human alveolar epithelial cells. The results of this study indicate that nicotine treatment enhances *S. aureus* biofilm formation by promoting initial attachment and extracellular DNA release but inhibits the virulence of this bacterium.

## Introduction

Chronic rhinosinusitis (CRS) is an inflammatory condition affecting the nose and nasal sinuses with a high worldwide prevalence^1^. Although CRS represents a considerable health burden and causes a significant reduction in the quality of life, the treatment strategies for CRS are still limited. This is partly because the mechanisms underlying the disease pathology are not well understood. Recently, the contribution of chronic bacterial infections involving biofilms in CRS pathology has been recognized^2^.

Biofilms are communities of bacteria retained within a microbial-derived matrix, which facilitates their survival. Mature biofilms are composed of bacteria, extracellular polysaccharide, extracellular DNA (eDNA), and proteins^3^. Given their high degree of resistance to the human immune system and the latest antibiotics, bacterial biofilms play an important role in the pathogenesis of many chronic human infections^4^. In 2004, Palmer *et al*. first reported the existence of biofilms on the sinus mucosa of patients with recalcitrant CRS^5^. Numerous studies have subsequently indicated the possible role of bacterial biofilms in CRS.

Tobacco smoke is an important threat to global health^6^. Despite intensive public health interventions, smoking rates are still very high worldwide^7^. Tobacco smoke has been reported to be correlated with CRS and poor sinus surgery outcomes^8,9^. However, whereas the impact of tobacco smoke on the human body has been studied extensively, the impact of smoke on the microbiome has been relatively less well studied. Increasing evidence indicates that tobacco smoke augments biofilm formation in multiple pathogenic bacteria^10–13^. In our previous study, we also demonstrated that cigarette smoke can enhance bacterial biofilm formation in multiple bacterial strains isolated from CRS patients^14^. However, our understanding of tobacco-induced bacterial biofilms is currently inadequate^15^.

*Staphylococcus aureus* is a common pathogen that plays a vital role in the condition of CRS patients due to its virulence and its ability to form biofilms^16^. Here, we examined the effect of nicotine—one of the most important components of tobacco—on *S. aureus* biofilm formation and virulence-related gene expression. Furthermore, we studied the mechanisms underlying this effect. The findings of this research will potentially contribute to enhancing our knowledge of tobacco smoke-induced bacterial biofilm formation and provide important information for developing novel therapeutic approaches for CRS.

## Results

### Nicotine enhances *S. aureus* biofilm formation

**Figure 1:**
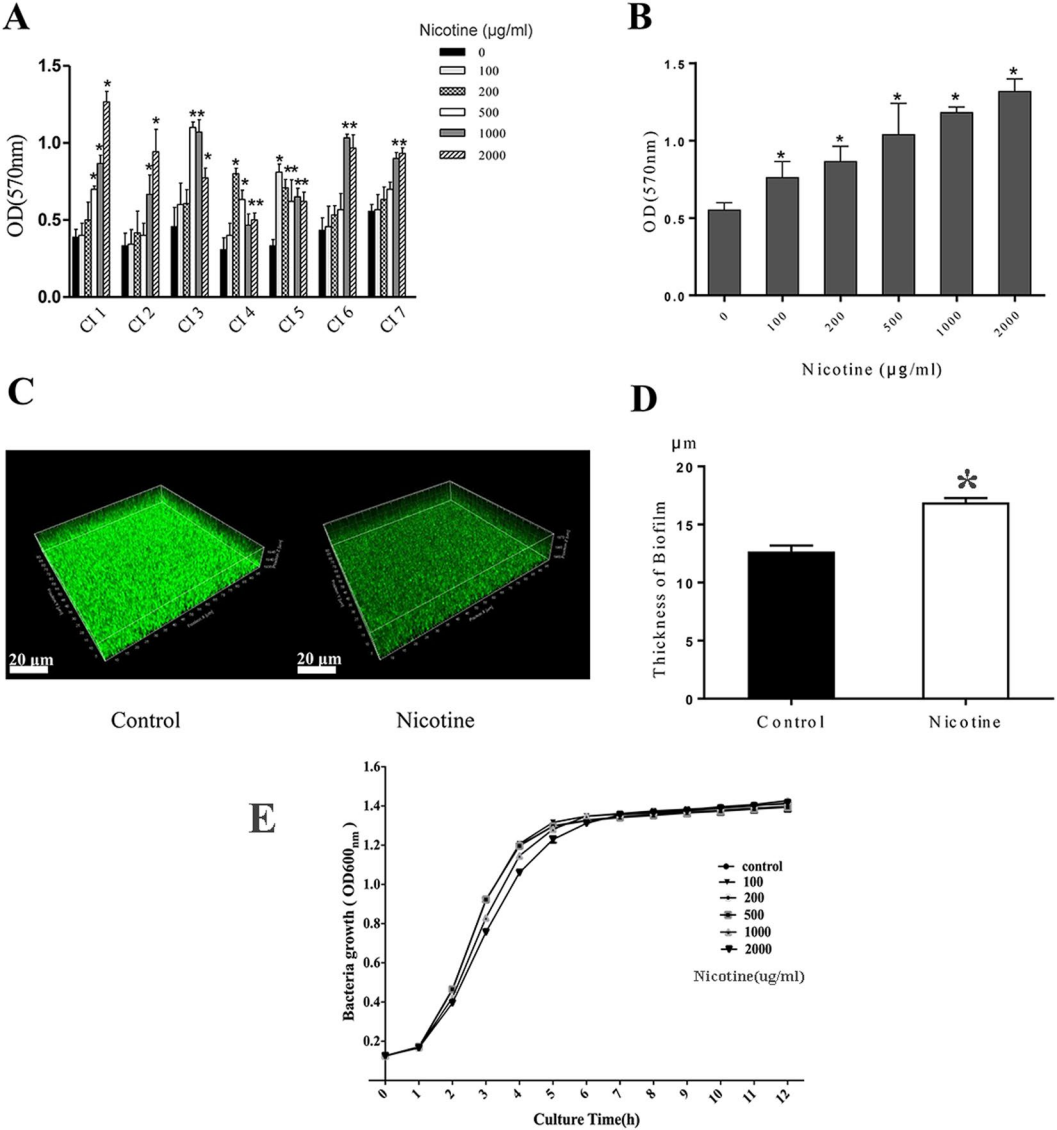
Nicotine induced biofilm formation in *Staphylococcus aureus*. (**A**) Clinical *S. aureus* strains were exposed to various concentrations of nicotine (0, 0.1, 0.2, 0.5, 1, 2 mg/mL). Crystal violet staining of biofilm-associated biomass revealed that the biofilm formation was increased in all strains in response to nicotine treatment. (**B**) A dose-dependent increase in biofilm formation was observed in USA300. Maximal biofilm formation was observed at 2 mg/mL. (**C**) Representatives of confocal images showed that denser biofilms were formed in the nicotine-treated group than in the control group. (**D**) The average thickness of biofilms in nicotine treatment group was 16.90 ± 0.66 μm (n = 3), while the thickness in the untreated group was 12.67 ± 0.91 μm (n = 3) (*p* < 0.05). (**E**) *S. aureus* USA300 strain FPR3757 growth curves under different concentrations of nicotine (0, 0.1, 0.2, 0.5, 1, 2 mg/mL) were determined by measuring the OD at a wavelength of 600 nm at 1-h intervals for 12 h. *P < *0.05* compared with untreated group. CI, clinical isolates.

In order to determine the influence of nicotine on *S. aureus* biofilm formation, we examined the effects of nicotine on seven biofilm-positive clinical *S. aureus* strains collected from the middle meatus of CRS patients as well as that of USA300 strain FPR3757. After incubating bacteria with nicotine for 24 h, there was a significant increase in the amount of biofilm produced by all strains, as determined using a microtiter plate assay (OD_570_). A dose-dependent effect of nicotine on *S. aureus* biofilm formation was observed, and different clinical strains exhibited maximal increases in biofilm formation at different concentrations of nicotine (Fig. 1A). In the case of the USA300 strain, a dose-dependent increase in biofilm formation was observed between 100 μg/mL and 2 mg/mL nicotine, with a maximal increase at 2 mg/mL (Fig. 1B) and a subsequent decrease at higher concentrations (data not shown). Thus, based on these observations and the findings of previous studies^17,18^, a nicotine concentration of 2 mg/mL was used in all the subsequent experiments to examine the mechanisms underlying nicotine-induced biofilm formation and virulence expression. Confocal laser-scanning microscopy (CLSM) was used to investigate the effect of nicotine on USA300 biofilm formation by measuring the thickness of 24 h mature biofilms, representative images of which are shown in Fig. 1C. The confocal microscopy measurements of biofilm thickness revealed that a dense biofilm had formed in the nicotine-treated group (16.90 ± 0.66 μm, n = 3), compared with the control group (12.67 ± 0.91 μm, n = 3) (*p* < 0.05) (Fig. 1D). Furthermore, we analyzed the effects of nicotine on *S. aureus* growth in response to different concentrations, and accordingly found that higher concentrations promoted a slower growth rate in the exponential phase (Fig. 1E).

### Nicotine treatment strengthens *S. aureus* initial attachment but has no effect on poly-*N*-acetylglucosamine polysaccharide synthesis

**Figure 2:**
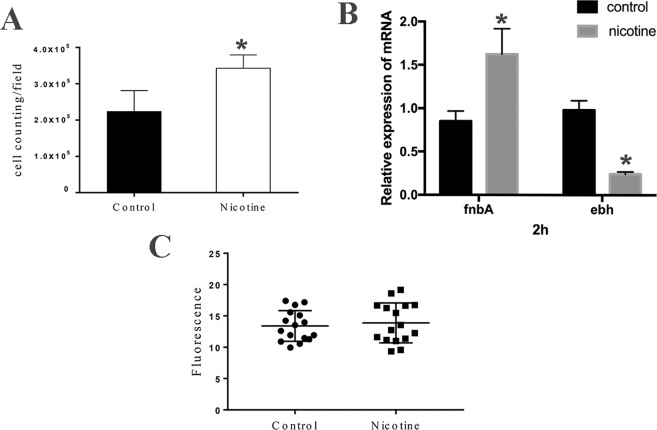
Nicotine increased *S. aureus* attachment and polysaccharide intercellular adhesin (PIA) production. (**A**) The number of attached bacteria was counted and analyzed. More attached cells were observed in the nicotine-treated group (3.44 ± 0.36 × 10^5^, n = 3) than in the control (2.25 ± 0.57 × 10^5^, n = 3). (**B**) Transcriptional levels of the *fnbA* and *ebh* genes in *S. aureus* USA300 strain cultured in TSB and TSB supplemented with 2 mg/mL nicotine for 2 h were detected by qRT-PCR(n = 3). (**C**) The levels of PIA were detected by CLSM. No significant difference between the nicotine treatment and control groups. *P < *0.05* compared with untreated group.

Bacterial attachment is the initial step of biofilm formation, and in this regard, our results indicated that after treatment with 2 mg/mL nicotine, a larger number of cells of USA300 strain FPR3757 had adhered to the bottom of polyethylene wells (3.44 ± 0.36 × 10^5^, n = 3), compared with the untreated control cells (2.25 ± 0.57 × 10^5^, n = 3) (*p* < 0.05) (Fig. 2A). Fibronectin-binding protein A (FnbA), which is a key surface-attached proteins and ECM-binding protein homologue (Ebh), a giant cell wall-related protein associated with virulence, are both key surface proteins, and therefore we examined the transcription level of these genes during the initial period of biofilm formation (2 h) in response to nicotine treatment. The results indicated that expression of the *fnbA* gene was increased whereas that of *ebh* was decreased after 2 h compared with the control group. (Fig. 2B) Furthermore, to examine the effects of nicotine on bacterial intercellular adhesion, we determined the synthesis of poly-*N*-acetylglucosamine polysaccharide (PIA) by CLSM. Observations indicated that the levels of PIA did not differ significantly between the nicotine-treated and non-nicotine-treated groups (Fig. 2C).

### Nicotine promotes the autolysis of *S. aureus*

**Figure 3:**
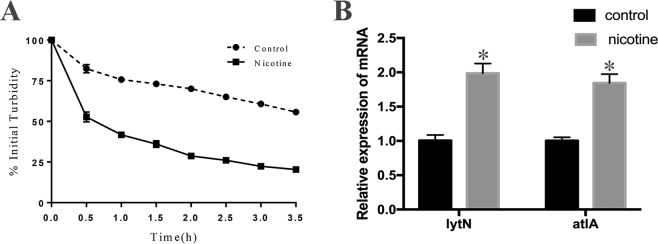
Nicotine increased *S. aureus* autolysis rate. (**A**) The mid-log phase culture (OD_600_ = 0.6) was collected and then resuspended in the same volume of 0.05 M Tris-HCl (pH 7.2) containing 0.05% Triton X-100, the solution was incubated at 30 °C and OD_600_ was measured every 30 min. The rate of autolysis was measured as the decline in optical density. (**B**) Transcriptional levels of the autolysis-related genes *lytN* and *atlA* in *S. aureus* USA300 strain cultured in TSB and TSB supplemented with 2 mg/mL nicotine detected by qRT-PCR(n = 3). Data were represented as mean ± SD of three independent experiments. *P < *0.05* compared with untreated group.

A Triton X-100-induced autolysis assay was performed to determine the effect of nicotine on autolysis of the *S. aureus* USA300 strain. As shown in Fig. 3A, the bacterial cultures treated with 2 mg/mL nicotine exhibited a significantly higher rate of autolysis than the control group culture. Consistently, the expression of autolysis-related genes, including *lytN* and *atlA*, were found to be upregulated in the nicotine-treatment group (Fig. 3B).

**Figure 4:**
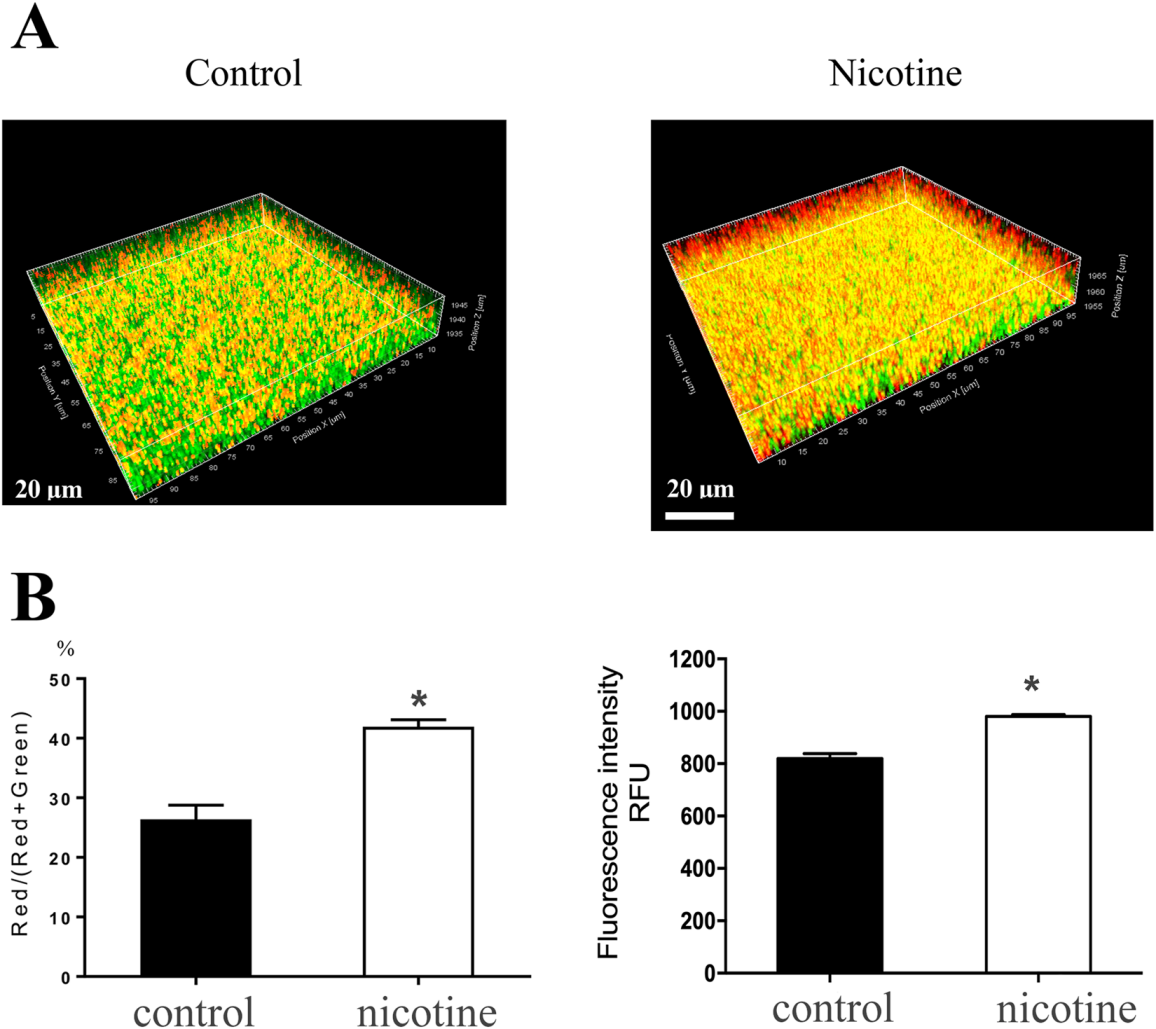
More dead cells were observed after nicotine treatment. Biofilms were grown for 24 h with or without 2 mg/mL nicotine(n = 3), then stained with SYTO9 (green fluorescence) and PI (red fluorescence) to represent the live and dead bacteria independently. (**A**) Biofilm were observing using CLSM with a 63 × 1.4-NA oil immersion objective. (**B**) Fluorescence was quantified using Leica Application Suite 1.0 software. A higher overall green fluorescence intensity was observed in the nicotine treated group. *P < 0.*05*. RFU, Relative fluorescence units.

Next, we performed CLSM to examine the 24 h biofilms of nicotine-treated USA300 cells, using LIVE/DEAD staining, the viable cells of which were stained by green fluorescence (SYTO9) and dead cells by red fluorescence (PI). A merged image of the two staining patterns is shown in Fig. 4A. The ratio of red fluorescence intensity to total fluorescence intensity was 41.94% ± 2.00% and 26.39% ± 4.09% (n = 3, *p* < 0.05) in the nicotine-treatment group and the control group, respectively; while the overall green fluorescence intensity in the two groups was 980.3 ± 12.34 and 819.3 ± 33.35 (*p* < 0.05), respectively (Fig. 4B).

### Nicotine facilitates eDNA release by *S. aureus*

**Figure 5:**
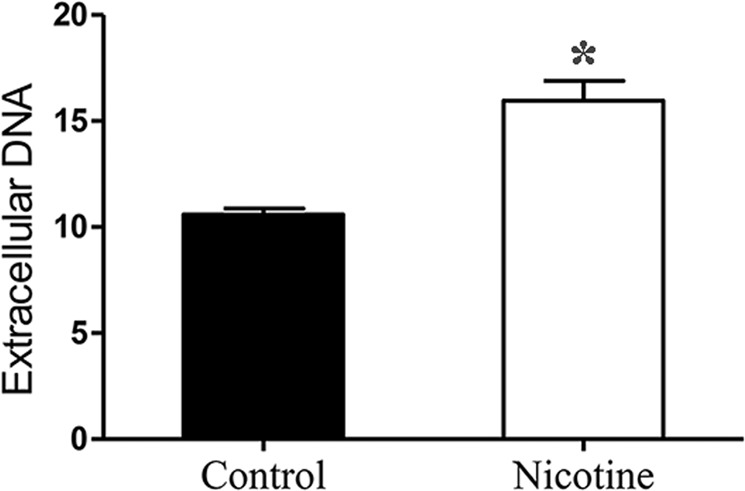
Nicotine enhanced the release of extracellular DNA (eDNA). The amount of eDNA in the matrix of *S. aureus* biofilm(24 h) were determined by measuring the fluorescence of PI-bound eDNA with the excitation/emission wavelength at 535/610 nm. Relative amount of eDNA was expressed as the fluorescence intensity per OD_600_ unit. The amount of eDNA was higher in the nicotine treatment group (15.95 ± 1.64, n = 3) as compared to the control group (10.59 ± 0.51, n = 3) *P < *0.05*.

The upregulation of autolysis is invariably associated with a higher release of eDNA. Thus, we evaluated the eDNA in 24 h *S. aureus* biofilms in the presence and absence of nicotine by staining with PI. The results accordingly revealed that the amount of eDNA (indicated by fluorescence intensities at a wavelength of 610 nm per OD_600_) was higher in the nicotine treatment group (15.95 ± 1.64, n = 3) compared with the control group (10.59 ± 0.51, n = 3) (*p* < 0.05) (Fig. 5).

### DNase I and Proteinase K inhibit nicotine-induced biofilm formation

**Figure 6:**
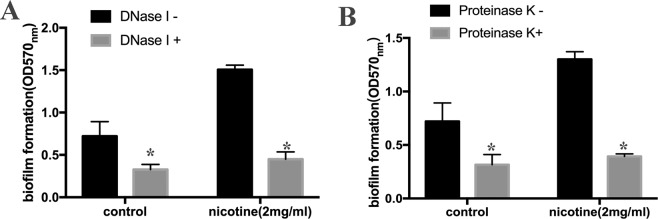
DNase I and Proteinase K inhibit nicotine-induced biofilm formation. The biofilm formation of *S. aureus* USA300 strain was detected using a microtiter plate assay by measuring crystal violet stained biofilm at OD570. DNase I (25 U/well) and Proteinase K (2 ug/ml) was added to the well in both 2 mg/ml nicotine treatment group and control group. (**A**) Treated by DNaseI, the average thickness of biofilms varied from 1.506 ± 0.04 to 0.450 ± 0.07 in the nicotine-treated group (n = 3) and from 0.720 ± 0.14 to 0.328 ± 0.05 in untreated group. (n = 3) (**B**) Proteinase K(2 ug/ml) disrupted biofilm formation, in the nicotine treated group decreasing from 1.300 ± 0.06 to 0.393 ± 0.02 (n = 3), and in the control group decreasing from 0.721 ± 0.14 to 0.316 ± 0.08 (n = 3). *P < *0.05*.

To evaluate whether enhanced biofilm formation is associated with an increase in eDNA release, we observed the effect of DNase I on biofilm formation in the USA300 strain with or without 2 mg/mL nicotine treatment. The results showing that OD_570_ was reduced from 1.506 ± 0.04 to 0.450 ± 0.07 in the nicotine-treated group and from 0.720 ± 0.14 to 0.328 ± 0.05 in untreated group, indicating that nicotine-induced biofilm formation is sensitive to DNase I (25 U/well) (Fig. 6A).

Furthermore, we also investigated whether the enhanced biofilm formation induced by nicotine can be inhibited by Proteinase K (2 μg/mL), and accordingly found that Proteinase K has a suppressive effect on biofilm formation, with OD_570_ values in the nicotine treated group decreasing from 1.300 ± 0.06 to 0.393 ± 0.02 (n = 3, *p* < 0.05), and those in the control group decreasing from 0.721 ± 0.14 to 0.316 ± 0.08 (n = 3, *p* < 0.05) (Fig. 6B)

### Nicotine treatment suppresses *S. aureus* virulence

**Figure 7:**
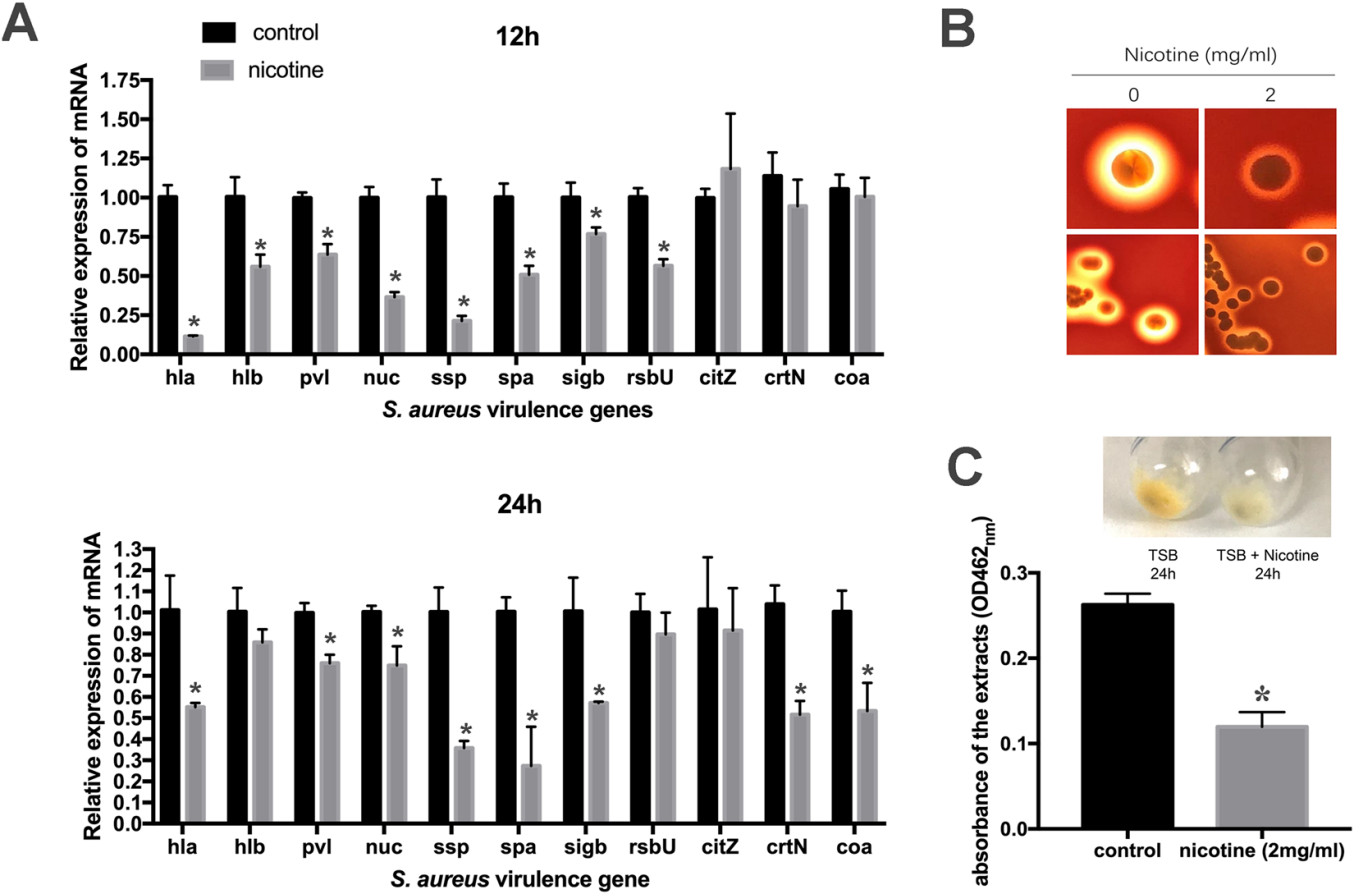
Nicotine treatment suppresses *S. aureus* virulence. (**A**) Transcriptional levels of the virulence genes in *S. aureus* USA300 strain cultured in TSB and TSB supplemented with 2 mg/mL nicotine for 12 h/24 h were detected by qRT-PCR(n = 3). In the nicotine-treatment group, the expression of the virulence genes, *hla*, *hlb*, *pvl*, *nuc*, *ssp*, *spa*, *sigB* was downregulated at both 12 h and 24 h, while expression of the coagulase gene *coa* and the pigmentation-related gene *crtN* was downregulated at 24 h. (**B**) The *S. aureus* USA300 strain was inoculated on normal blood agar plates (left) and blood agar plates containing 2 mg/mL nicotine (right), respectively. After incubation at 37 °C for 24 h, a smaller β-hemolytic ring (β-hemolytic phenotype) was formed on the blood agar plate containing nicotine than the control. (**C**) Nicotine treatment suppressed carotenoid pigment formation. The *S. aureus* USA300 strain was cultured for 24 h in TSB (left) and TSB supplemented 2 mg/mL with nicotine (right) respectively. The pigment formed by bacterial cell pellets in nicotine group was reduced as compared to the control. Pigment production was quantified at OD_462_, decreasing from 0.263 ± 0.01 in the control group (n = 3) to 0.119 ± 0.01 in the nicotine-treated group (n = 3). These photos are representative of three independent experiment.

As one of the major human pathogens, *S. aureus* can produce numerous virulence factors, including alpha hemolysin (*hla*) and beta hemolysin (*hlb*), responsible for hemolysis; nuclease (*nuc*), serine protease (*ssp*), and surface proteins A (*spa*), and genes responsible for pigmentation, including *sigB, rsbU* (global regulators), *citZ*, and *crtN*^19^. We examined the effects of nicotine on the expression levels of these virulence genes in the *S. aureus* USA300 strain using qRT-PCR. The results showed that in the nicotine treatment group, expression of the virulence-related genes *hla*, *hlb*, *pvl*, *nuc*, *ssp*, *spa*, and *sigB* was downregulated at both 12 h and 24 h, the expression of *rsbU* decreased at 12 h, whereas only the coagulase gene *coa* and the pigmentation-related gene *crtN* was downregulated at 24 h (Fig. 7A).

To investigate whether the altered gene expression patterns affected the virulence-related phenotypes, the hemolysis (24 h) and carotenoid pigmentation formation (24 h) of USA300 cells were investigated in the presence and absence of nicotine (2 mg/mL). As expected, the hemolysis and pigmentation phenotypes were altered upon treating with nicotine. A clear/complete hemolytic ring (β-hemolytic phenotype) was observed around USA300 colonies on blood agar plates, whereas a smaller β-hemolytic ring was formed on the blood agar plate containing nicotine (Fig. 7B). Compared with the control cells, USA300 cells cultured in TSB medium supplemented with 2 mg/mL nicotine for 24 h showed visually reduced pigment production, whose absorbance at OD_462_ decreased from 0.263 ± 0.01 in the non-nicotine treated group to 0.119 ± 0.01 in the nicotine-treated group (n = 3, *p* < 0.05), thereby indicating that nicotine suppresses bacterial virulence (Fig. 7C).

### Nicotine attenuates the capacity of *S. aureus* to invade epithelial cells

**Figure 8:**
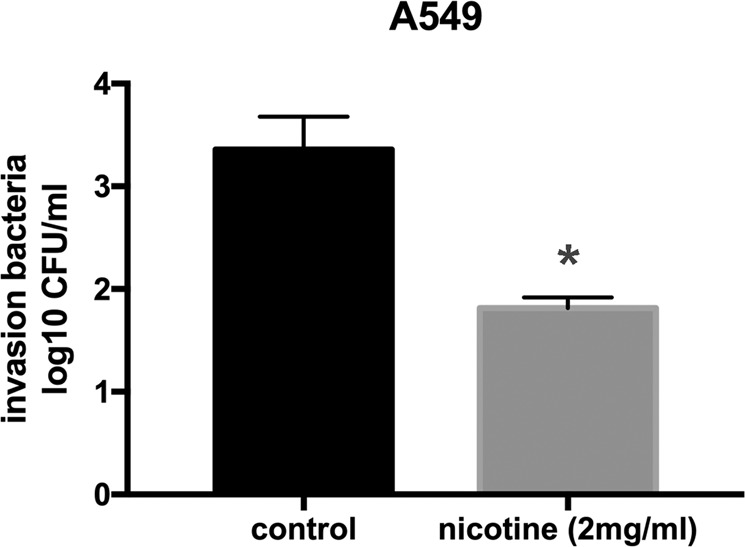
Nicotine treatment inhibits *S. aureus* invasion in A549 epithelial cells. Cell monolayers was infected with a bacterial multiplicity of infection (MOI) of 10:1. After 2 h of incubation, 200 μg/mL Gentamicin and 40 μg/mL Lysostaphin was added to kill the bacteria external to the monolayer cells at 37 °C in 5% CO_2_ for 30 min. Then the monolayer cells were subsequently lysed with 200 μL 0.1% Triton X-100 for 20 min at room temperature, and were then diluted with PBS and plated on TSB-agar plates to determine the number of intracellular bacteria. Intracellular CFUs in nicotine treated group and control group were 3.360 ± 0.25 log^10^ and 1.816 ± 0.08 log^10^ respectively. (n = 3, *p* < 0.05) Data were represented as mean ± SD of three independent experiments. *P < *0.05*.

Epithelial cells are the first-line defense against disease-causing organisms, and ability of bacteria to invading these cells is a facet reflecting the virulence of these organisms. To further elucidate the effect of nicotine on the virulence of *S. aureus*, we investigated the capacity of *S. aureus* to invade A549 human alveolar epithelial cells. Our results showing CFUs of 3.360 ± 0.25 log^10^ and 1.816 ± 0.08 log^10^ (n = 3, *p* < 0.05) for the control and nicotine-treated groups intracellular respectively, indicated that nicotine inhibited the ability of these bacteria to invading A549 cells (Fig. 8).

### Nicotine affects biofilm formation in *S. aureus* in an accessory gene regulator-dependent manner

**Figure 9:**
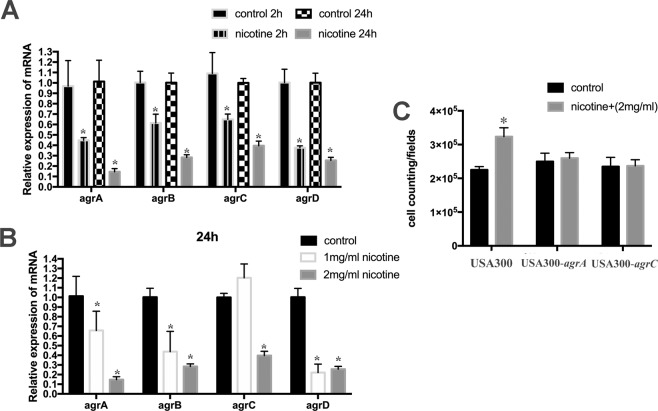
Agr system plays a role in biofilm development induced by nicotine. (**A**) The relative mRNA expression of Agr system genes (*agrA, agrB, agrC*, and *agrD*) was evaluated at 2 h and 24 h during the biofilm formation process in the presence or absence of nicotine. (**B**) The relative mRNA expression of *agrA, agrB, agrC*, and *agrD* genes were detected when treated with 0, 1 mg/ml, 2 mg/ml nicotine at 24 h. (**C**) The number of attached bacteria was counted and analyzed in USA300, *USA300-agrA*, *USA300-agrC*. The nicotine-induced enhancement of the initial attachment stage was abolished in these two mutant strains. Data were represented as mean ± SD of three independent experiments. *P < *0.05*.

Previous studies have indicated that the activity of the accessory gene regulator (Agr) system appears to have an influence on *S. aureus* virulence and enhance biofilm formation by this bacterium^20–22^. In this study, we explored the expression of Agr system genes (*agrA, agrB, agrC*, and *agrD*) at 2 h and 24 h during the biofilm formation process in the presence or absence of nicotine. The results showed that expression of *agrA, agrB, agrC*, and *agrD* in the nicotine-treated group were lower than those in the control group, (n = 3, *p* < 0.05), and that the expression appeared to be both time- and dose-dependent. Figure 9A shows that gene expression gradually decreased from 0 h to 2 h and 2 h to 24 h. Furthermore, at 24 h, we found that the degree of reduction in the 2 mg/mL group was more pronounced than that in the 1 mg/mL group (Fig. 9B).

In addition, we investigated whether the strengthened initial attachment due to nicotine exposure was a result of Agr dysfunction. By observing the effect of nicotine on USA300 FPR3757 mutants harboring transposon insertions in the *agrA* and *agrC* genes, we found that nicotine-induced enhancement of the initial attachment stage was abrogated in these two mutant strains, indicating that the Agr system is involved in the nicotine-potentiated initial attachment process (Fig. 9C).

## Discussion

Given that *S. aureus* is a frequent colonizer of the nasal mucosa and lower respiratory tract passages, it will be exposed to cigarette smoke during smoking. Previous studies have indicated that an extract of cigarette smoke induces biofilm formation in various bacterial species and that nicotine enhances biofilm formation by *Streptococcus mutants*, *Streptococcus gordonii* and *Staphylococcus epidermidis*^17,23–25^. In the present study, we found that nicotine can enhance *S. aureus* biofilm formation capability in both clinical *S. aureus* strains and USA300 strain FPR3757. We hypothesize that the nicotine-induced enhancement of *S. aureus* biofilm formation may be a general phenomenon and that it might represent a protective reaction on the part of *S. aureus* to adverse environments, such as exposure to nicotine. Under adverse conditions, *S. aureus* develops a defense phenotype biofilm by altering gene expression, which results in the bacteria becoming embedded in a protective biofilm that renders them less susceptible to eradication.

In the current study, we found that nicotine treatment enhances *S. aureus* biofilm formation by increasing initial attachment and the release of extracellular DNA, although it also attenuates bacterial virulence, including inhibition of the expression of major virulence-related genes, reducing pigment production, and reducing bacterial invasion of epithelial cells, the latter of which is a pathological process related to activity of the Agr system.

Nicotine is one of the most toxic chemicals in tobacco. Huang *et al*.^17^ mentioned that high levels of nicotine of up to 2.3 mg/mL are detected in the saliva of smokers. Although to date there have been no data published regarding nicotine concentrations in nasal mucus, several facts indicate that these concentrations could be similar to the levels of nicotine in saliva. Firstly, the nose is one of the most exposed organs to smoke and the nasal mucosa is one of the major routes via which nicotine is absorbed from tobacco^26^. Secondly, the nasal and oral cavities are interconnected, and are hence simultaneously exposed to nicotine during smoking. Thus, levels of nicotine in nasal mucus may reach concentrations in the range of that which we used in the present study (2 mg/mL) to investigate the underlying mechanism of nicotine-induced biofilm formation and virulence expression *in vitro*.

Biofilm formation proceeds in two steps, namely, an initial bacterial adhesion followed by cell proliferation and aggregation^27^. The essential step in the establishment of any *Staphylococcus* infection is the attachment to host tissues. We observed that a larger number of bacteria were attached to the polyethylene surface of culture plate wells in a nicotine-treated group than in a control group, thereby indicating that nicotine may enhance the initial step in *S. aureus* biofilm formation. These results are consistent with the findings of Cogo *et al*., who demonstrated that cotinine, the predominant nicotine metabolite, enhances the adhesion of *Porphyromonas gingivalis* to epithelial cell monolayers^28^. Surface proteins are the main components involved in biofilm formation, particularly during initial attachment^29,30^. *FnbA* is a well-known surface protein associated with the attachment stage of biofilm formation and Ebh is a giant protein found on the surface of *S. aureus*. In infected mice, *ebh* variants have been shown to be associated with the diminished virulence of *S. aureus*^31^. In response to nicotine treatment(2 h), we found that transcription levels of *fnbA* were increased, whereas the expression of *ebh* decreased. Furthermore, expression of the autolysis-related gene *atlA*, which is also associated with initial attachment, was also found to be increased.

The staphylococcal biofilm matrix has been reported to contain eDNA proteins, and polysaccharides, and we found that compared with the control group, larger amounts of eDNA were present in the matrix of *S. aureus* biofilms in the nicotine-treated group. Consistent with the findings of Kulkarni *et al*.^10^, we observed that the enhanced biofilm formation promoted by nicotine was suppressed by DNase I and proteinase K, thereby providing evidence that eDNA and proteins may play key roles in the nicotine-induced enhancement of initial attachment. Cell autolysis is a major source of eDNA in biofilms and a previous study has demonstrated that such autolysis could have a significant impact on biofilm formation^32,33^. Thus, we speculated that nicotine might increase *S. aureus* autolysis. However, results from previous studies to determine the effect of CS on bacterial autolysis are not always consistent: MacEachern^34^ observed that exposure of *S. aureus* USA300 to CS resulted in an increased resistance to Triton-induced lysis, as a consequence of cell wall alteration, including changes in surface charge and hydrophobicity, Kulkarni *et al*. found DNase I inhibited CS-induced biofilm formation and observed 6-fold increase of autolysis associated gene, *cidA*, in USA300. We found that nicotine significantly increased *S. aureus* autolysis compared with the control group.

It has previously been shown that *S. aureus* biofilm formation is tightly regulated by a quorum sensing system, accessory gene regulator (Agr*)* system^20^, encoded by *agrBDCA* operon. The *agrC/agrA* two-component system negatively regulates *S. aureus* biofilm formation, partially by increasing extracellular protease production and inhibiting cell adhesion to surfaces. Our real-time PCR analysis revealed that transcription of the *agr* operon (*agrA, agrB, agrC*, and *agrD*) showed a marked decrease in expression in the nicotine treatment group (at 2 h and 24 h) depending on nicotine concentration and time exposure. Furthermore, we found that in USA300 mutants harboring a transposon insertion in either the *agrC* or *agrA* gene, the nicotine-induced increase in initial attachment was abolished. Taken together, our observations indicated that the augmentation of biofilm formation by nicotine may be directly or indirectly related to downregulation of the Agr system.

As one of the major human pathogens, *S. aureus* can produce numerous virulence factors, whose expression is influenced by the microenvironment in the human body and is related with the process of bacterial pathogenesis. For example, it has been reported that α-toxin (α-hemolysin), a major cytolytic toxin expressed by the majority of *S. aureus* clinical isolates, is associated with the persistence of *S. aureus* cells in tissues and can also provoke cellular responses in nasal polyp cells from CRS patients, therefore, may play a role in the pathophysiology of CRS^35^. Although excess expression of virulence factors including exotoxins may enhance bacterial pathogenicity in the host, it can trigger augmented immune responses, which may lead to acute inflammations, hypersensitivity and then quick clearance of bacteria cells. Thus, the transcriptional regulation of virulence genes is vital for bacterial survival and adaption in the host. In *S. aureus*, the Agr system initiates RNAIII transcription and plays a pivotal role in the regulation of myriad virulence factors, especially exotoxin genes^36^. A recent extensive study conducted by Lacoma *et al*.^29^ showed that the Agr system mediates CS-induced biofilm augmentation and CS-reduced toxin production. Based on the data in this study and in the literatures, we hypothesize that the presence of nicotine represses the expression of *agrBDCA* operon and thereby may also have an inhibitory effect to various virulence-related genes. In line with the expectation, the virulence genes including *hla* (alpha hemolysin), *hlb* (beta hemolysin), *pvl* (leucocidin), *nuc* (thermostable nuclease), *ssp* (serine protease), *spa* (protein A) and the genes responsible for pigmentation, including *sigB, rsbU, citZ*, and *crtN*^19^, showed decreased transcriptional levels in the nicotine treatment group at 12 h or 24 h. Consequently, nicotine treatment inhibited subsequent β-hemolysis on blood agar plates and carotenoid pigment production. Furthermore, using the A549 cell line to assess the invasion ability of *S. aureus* after nicotine treatment, we showed that nicotine decreased the number of intracellular bacteria. We speculate that on one hand, nicotine induces reduction in multiple bacterial virulence genes expression and may hinder the progress of acute infection. On the other hand, it enhances bacterial biofilm formation, which can protect bacteria from the host immune attack and therefore contribute to the persistence of this pathogen.

In conclusion, the results of this study provide further insights into the mechanisms whereby tobacco smoke induces *S. aureus* biofilm formation. We observed that nicotine can enhance biofilm formation in both clinical *S. aureus* strains obtained from CRS patients and the USA300 FPR 3757 strain. The altered biofilm formation was associated with enhanced initial cell attachment, elevated eDNA release, and enhanced *S. aureus* autolysis. Additionally, we found that nicotine repressed transcription of various virulence-related genes and inhibited β-hemolysis on blood agar plates, carotenoid pigment production and invasion in A549 cells by *S. aureus*. We hypothesize that the nicotine-induced reduction in bacterial virulence and enhancement of biofilm formation increases bacterial fitness and strengthens adaption to the harsh nasal environment, thereby may contribute to chronic infection.

## Materials and Methods

### Ethics statement

All procedures performed in studies involving human participants were in accordance with the ethical standards of the Institutional Review Board of Eye & ENT Hospital (reference number KJ2011-31) and with the 1964 Helsinki Declaration and its later amendments or comparable ethical standards. In addition, informed consent was obtained from all participants.

### Bacteria strains and culture media

This study was previously approved by the Ethics Committee on Research of Eye & ENT Hospital. The clinical strains of *S. aureus* were collected during endoscopic surgery performed at the Shanghai Eye and ENT Hospital of Fudan University, and isolated from the middle meatus of CRS patients. The bacterial strains used in this study are listed in Table 1. *S. aureus* USA300 strain FPR3757 and mutants of this strain harboring transposon insertions in the *icaA*, *ebh, agrA*, and *agrC* genes were provided by Professor Ying Zhang at Johns Hopkins University. Tryptone soya broth (TSB; Oxoid, USA) was used for bacterial culture, and TSB supplemented with 1% glucose (TSBG) was used for bacterial biofilm formation experiments.

**Table 1:** Bacterial strains used in this study.

Stains	Description	Source or Reference
USA300 FPR3757	a MRSA strain (GenBank Accession Number: NC 007793)	^39^
USA300-*icaA*	USA300 FPR3757 with a transposon insertion in the *icaA* gene	This study
*USA300-agrA*	USA300 FPR3757 with a transposon insertion in the *agrA* gene	This study
*USA300-agrC*	USA300 FPR3757 with a transposon insertion in the *agrC* gene	This study

### Detection of bacterial biofilm formation

Bacterial strains were grown in TSB with or without nicotine for 6 h at 37 °C to obtain bacteria in a mid-exponential phase. The cultures were then diluted 1:200 with TSBG supplemented with or without nicotine, and 200 μL of bacterial suspension was added to each well of a 96-well microplate and incubated at 37 °C for 24 h. To determine the effect of DNase I and proteinase K on biofilm formation, 5 μL DNase I (5 U/μL, Takara, Shanghai, China) and 2 μg/mL Proteinase K (Sango, Shanghai, China) were added to the wells. Thereafter, the wells were washed three times with phosphate-buffered saline (PBS) to remove unattached bacteria and then 200 μL of 100% methanol was added to each well to fix the attached cells at room temperature for 20 min. After removal of the methanol, the biofilms were air-dried and stained with 2% crystal violet at room temperature for 8 min. The wells were then washed with running tap water until the water was clear. Subsequently, 200 μL of 10% acetic acid was added to each well and incubated for 1 h. Finally, the stained biofilms were quantified by estimating the optical density (OD) at 570 nm using a microtiter-plate reader (DTX 880 Multimode Detector; Beckman Coulter, USA). The *S. aureus* USA300 strain and its isogenic *icaA* gene transposon insertion mutant were used as a biofilm-forming strain and a non-biofilm-forming control strain, respectively.

### Bacterial growth curve determination

*S. aureus* growth curves were determined by measuring the OD at a wavelength of 600 nm using an automated growth curve detector (Biocreen C, Finland). Briefly, overnight cultures were diluted (1:200) and incubated in the presence of different nicotine concentration with shaking at 220 rpm and 37 °C. The OD_600_ values of bacterial cultures were measured at 1h intervals for 12 h.

### Observation of *S. aureus* biofilms by confocal laser-scanning microscopy (CLSM)

*S. aureus* biofilms were cultured in TSB with or without 2 mg/mL nicotine in glass-bottomed dishes, washed with three times with PBS, and then stained with LIVE/DEAD staining dye [1 μM of SYTO9 and 1 μM of propidium iodide (PI)] for 20 min. The biofilms were observed using a confocal laser-scanning microscope (Leica TCS SP8; Leica Microsystems, Germany) with a × 63 1.4-NA oil immersion objective. Fluorescence was quantified using Leica Application Suite 1.0 software (Leica Microsystem, Germany), and IMARIS 7.0 software (Bitplane, USA) was used to generate three-dimensional images of biofilms.

### Initial bacterial attachment assays

Bacteria strains were cultured in TSB with or without 2 mg/mL nicotine to mid-exponential phase, and the bacterial cells were diluted with TSB to OD_600_ = 0.1. The diluted culture was added to cell culture-treated 6-well polystyrene microtiter plates (Nunc, Denmark; 1 mL per well) and incubated at 37 °C for 2 h. Thereafter, the attached cells were washed gently with PBS (three times) and imaged. For each sample, six representative optical fields were randomly selected, and cells were counted using ImageJ software.

### Detection of polysaccharide intercellular adhesin (PIA) by spectrofluorometric assay

*S. aureus* biofilms were formed with or without 2 mg/mL nicotine in 96-well polystyrene microplates, washed gently with PBS, and then stained with 200 μL of 5 μg/mL wheat germ agglutinin (WGA)-Alexa Fluor 350 fluorescent conjugate (ThermoFisher, USA). After incubation at 4 °C for 2 h in the dark, the conjugate was removed, and the wells were gently washed three times with PBS. The plate was then air-dried at room temperature for 15 min, following which, 200 μL of 33% acetic acid was added to each well. The biofilms in the wells were incubated at 37 °C for 1 h, scraped thoroughly, and then 150 μL solution from each well was transferred to a solid black microplate (PerkinElmer, USA) for top reading using a Varioskan^TM^ LUX microplate reader (ThermoFisher, USA; fluorescence at λ_excitation_ = 346 nm and λ_emission_ = 442 nm).

### Triton X-100-induced bacterial autolysis assay

To determine the effect of nicotine on *S. aureus* autolysis, a Triton X-100-induced autolysis assay was performed. An overnight culture of the *S. aureus* USA300 strain FPR3757 was diluted 1:200 with TSB containing 1 M NaCl, and was then grown with or without 2 mg/mL nicotine to mid-log phase (OD_600_ ~ 0.6). The cells were collected by centrifugation and washed twice in cold sterile PBS. After resuspension in the same volume of 0.05 M Tris-HCl (pH 7.2) containing 0.05% Triton X-100, the solution was incubated at 30 °C and OD_600_ values were measured at 30-min intervals. The autolysis rate was determined by calculating the decline in OD_600_ values. Data are represented as the mean ± SD of three independent experiments.

### Detection of extracellular DNA (eDNA)

The quantity of eDNA in *S. aureus* cultures was determined using modifications of the methods described by Allesen-Holm *et al*. and Qin *et al*.^37,38^. Briefly, *S. aureus* strains were cultured overnight in TSB supplemented with or without 2 mg/mL nicotine, following which. the cultures were diluted with minimal growth medium (AB medium) supplemented with 0.5% glucose, 10% TSB, and 0.05 mM PI, to an OD_600_ of 0.001. The diluted cultures were transferred to a 96-well microplate (200 μL per well) and incubated with or without 2 mg/mL nicotine at 37 °C for 24 h. The OD_600_ values were measured using a microplate reader (BioRAD, USA). The fluorescence of PI-bound eDNA was measured at excitation/emission wavelengths of 535/610 nm using a Varioskan™ LUX microplate reader. The relative amount of eDNA was expressed as the fluorescence intensity per OD_600_ units.

### RNA extraction

Overnight cultures of USA300 were diluted 1:200 with TSB and incubated with or without 2 mg/mL nicotine at 37 °C to an OD_600_ of 0.6 (mid-log phase). Bacterial cells were collected by centrifugation (4000 × *g*), washed three times with ice-cold saline, and homogenized using a Beadbeater-16 homogenizer (Biospec, USA). The bacterial RNA was purified using an RNeasy kit (Qiagen, Germany).

### Quantitative real-time PCR analysis

The primers used in this study are listed in Table 2. DNase-treated RNA was reverse transcribed (Takara) to cDNA. All samples were prepared in triplicate and then quantified by qPCR using an ABI 7500 real-time PCR system (Applied Biosystems, USA) and SYBR Green I mixture (Takara). The data were normalized using *gyrB* as an internal control.

**Table 2:** Primers used in this study.

Primers	Sequence (5′ → 3′)
gyrB-F	ACATTACAGCAGCGTATTAG
gyrB-R	CTCATAGTGATAGGAGTCTTCT
lytN-F	GACACCATTAGTAGAACCAA
lytN-R	AACATTGCCATCCATAAC
atlA-F	AAGTTGTTGTAGTTGATGATGA
atlA-R	TAGTAATACGATGTCTGGTTCT
hla-F	GGTATATGGCAATCAACTT
hla-R	CTCGTTCGTATATTACATCTAT
hlb-F	GCACTTACTGACAATAGT
hlb-R	GACTAACTAACTTCAAATCAG
pvl-F	TGCCAGTGTTATCCAGAG
pvl-R	ATTATTACCTATCCAGTGAAGTTG
nuc-F	GCGATTGATGGTGATACGGTTA
nuc-R	TTAGGATGCTTTGTTTCAGGTGTA
ssp-F	CGGTGTAGTTGTAGGTAA
ssp-R	TTGGATAATTGTCTTGGTTAA
spa-F	AGTGCTAACCTATTGTCAGA
spa-R	ACCATTGCGTTGTTCTTC
sigB-F	GATGAACTAACCGCTGAAT
sigB-R	TTCCATTGCTTCTAACACTT
rsbU-F	ATAACGATGGCACAATGA
rsbU-R	TGAGTGTCCATAAGAATCC
citZ -F	ACCAACAGATATAGAAGTAGAAG
citZ -R	ATGATGATACCGCACAAC
crtN -F	AATGCTGAACAAGAGTAATC
crtN -R	AGTGAATGGTGACATAAGA
coa -F	CTCAAGGAGAATCAAGTG
coa -R	AATGTTCCATCGTTGTATT
fnbA-F	TTCCTTAACTACCTCTTCT
fnbA -R	CAATCATATAACGCAACAG
ebh-F	GTCAGTCGCATCACCATT
ebh-R	CACAATCATCAATCCAAGCATAT
agrA-F	GCAGTAATTCAGTGTATGTTCA
agrA -R	TATGGCGATTGACGACAA
agrB-F	GGTGTAATCTCAGTATATGC
agrB-R	GCTTCTATTATGATGCCTAA
agrC-F	GCAGTATTGGTATTATTCTTGA
agrC -R	TGCGTGGTATATCATCAG
agrD-F	ACATCGCAGCTTATAGTA
agrD-R	CGTGTAATTGTGTTAATTCT

### Analysis of hemolytic phenotype

The *S. aureus* USA300 strain was inoculated on blood agar plates (BioMérieux, China) and blood agar plates containing 2 mg/mL nicotine. The plates were cultured at 37 °C for 24 h, after which the hemolytic phenotype was observed and photographed.

### Cell culture and epithelial cell invasion assay

A549 human alveolar epithelial cells (ATCC CCL185) were cultured in high-glucose Dulbecco’s modified Eagle’s medium (DMEM) (Hyclone, USA) supplemented with 10% fetal bovine serum (FBS, Gibco). One day before infection, approximately 1 × 10^5^ cells were seeded in each well of 24-well plates (Costar, USA), and incubated at 37 °C in a 5% CO_2_ atmosphere overnight. The cells were infected at a multiplicity of infection (MOI) of 10:1 and the 24-well plate was centrifuged at 500 × *g* for 3 min. The bacterial cell mixtures were then incubated at 37 °C in 5% CO_2_ for 2 h. Following incubation, each well was washed twice with 500 μL PBS and medium containing 200 μg/mL Gentamicin and 40 μg/mL Lysostaphin was added to kill the bacteria external to the monolayer cells. The plate was then incubated at 37 °C in 5% CO_2_ for 30 min, followed by twice washes with 500 μL PBS. The monolayer cells were subsequently lysed with 200 μL 0.1% Triton X-100 for 20 min at room temperature, and were then diluted with PBS and plated on TSB-agar plates to determine the number of intracellular bacteria (Colony-Forming Units, CFU).

### Measurement and of carotenoid pigment

Colonies of *S. aureus* USA300 were cultured in TSB or TSB supplemented with 2 mg/mL nicotine at 37 °C for 24 h with shaking. The bacterial cells were thereafter collected by centrifugation and washed three times with distilled water. The formation of carotenoid pigment by bacterial cells of the same wet weight was established visually.

### Quantification of carotenoid pigment

*S. aureus* USA300 cells were cultured in TSB or TSB supplemented with 2 mg/mL nicotine at 37 °C for 24 h with shaking. Cells were harvested by centrifugation (10000 × *g*, 2 min), and then washed twice with PBS, following which, 100% methanol was added to extract staphyloxanthin and carotenoids in a water bath at 55 °C for 5 min. The resulting methanol extract liquid was centrifuged and the supernatant containing carotenoid pigment was then quantified by estimating the OD at 462 nm using a microplate reader (BioRAD, USA).

### Data analysis

Data are presented as the mean ± standard deviation. Comparison between two groups was made using unpaired two-tailed *t*-tests. One-way ANOVA followed by Bonferroni’s post hoc test was applied to compare between three or more groups. Prism 5 (GraphPad Software, Inc, CA, USA) was used for statistical analysis. Statistical significance was defined as a two-tailed *P* value < 0.05.
